# Shifting human resources for health in the context of ART provision: qualitative and quantitative findings from the Lablite baseline study

**DOI:** 10.1186/s12913-016-1891-7

**Published:** 2016-11-16

**Authors:** Misheck J. Nkhata, Margaret Muzambi, Deborah Ford, Adrienne K. Chan, George Abongomera, Harriet Namata, Ivan Mambule, Annabelle South, Paul Revill, Caroline Grundy, Travor Mabugu, Levison Chiwaula, James Hakim, Cissy Kityo, Andrew Reid, Elly Katabira, Sumeet Sodhi, Charles F. Gilks, Diana M. Gibb, Janet Seeley, Fabian Cataldo, the Lablite team, F. Cataldo, A. K. Chan, L. Chiwaula, M. J. Nkhata, F. Mirimo, S. Kiwuwa, J. Seeley, G. Abongomera, C. Kityo, H. Namata, J. Hakim, T. Mabugu, M. Muzambi, A. Reid, S. Kaggwa, E. Katabira, I. Mambule, C. F. Gilks, P. Revill, D. Ford, D. M. Gibb, C. Grundy, S. Hoskins, S. Joseph, A. South, M. Thomason, I. Weller, Alfred Matengeni, Florence Chinguwo, Nwaka Mwambene, Kenneth Phiri

**Affiliations:** 1 Dignitas International, Zomba, Malawi; 2 University of Zimbabwe, Harare, Zimbabwe; 3 MRC Clinical Trials Unit at University College London, London, UK; 4 Division of Infectious Diseases, Sunnybrook Health Sciences Centre, University of Toronto, Toronto, Canada; 5 Joint Clinical Research Centre, Kampala, Uganda; 6 Infectious Diseases Institute, Makerere University, Mulago, Uganda; 7 Centre for Health Economics, University of York, York, UK; 8 Department of Family and Community Medicine, University of Toronto, Toronto, Canada; 9 Department of Family and Community Medicine, University Health Network, Toronto Western Hospital, Toronto, Canada; 10 School of Population Health, University of Queensland, St Lucia, Australia; 11 MRC/UVRI Uganda Research Unit of AIDS, Entebbe, Uganda

**Keywords:** Human Resources for Health, HIV, ART decentralisation, Task Shifting, Health Care Workers, Malawi, Uganda, Zimbabwe

## Abstract

**Background:**

Lablite is an implementation project supporting and studying decentralized antiretroviral therapy (ART) rollout to rural communities in Malawi, Uganda and Zimbabwe. Task shifting is one of the strategies to deal with shortage of health care workers (HCWs) in ART provision. Evaluating Human Resources for Health (HRH) optimization is essential for ensuring access to ART. The Lablite project started with a baseline survey whose aim was to describe and compare national and intercountry delivery of ART services including training, use of laboratories and clinical care.

**Methods:**

A cross-sectional survey was conducted between October 2011 and August 2012 in a sample of 81 health facilities representing different regions, facility levels and experience of ART provision in Malawi, Uganda and Zimbabwe. Using a questionnaire, data were collected on facility characteristics, human resources and service provision. Thirty three (33) focus group discussions were conducted with HCWs in a subset of facilities in Malawi and Zimbabwe.

**Results:**

The survey results showed that in Malawi and Uganda, primary care facilities were run by non-physician clinical officers/medical assistants while in Zimbabwe, they were run by nurses/midwives. Across the three countries, turnover of staff was high especially among nurses. Between 10 and 20% of the facilities had at least one clinical officer/medical assistant leave in the 3 months prior to the study. Qualitative results show that HCWs in ART and non-ART facilities perceived a shortage of staff for all services, even prior to the introduction of ART provision. HCWs perceived the introduction of ART as having increased workload. In Malawi, the number of people on ART and hence the workload for HCWs has further increased following the introduction of Option B+ (ART initiation and life-long treatment for HIV positive pregnant and lactating women), resulting in extended working times and concerns that the quality of services have been affected. For some HCWs, perceived low salaries, extended working schedules, lack of training opportunities and inadequate infrastructure for service provision were linked to low job satisfaction and motivation.

**Conclusions:**

ART has been decentralized to lower level facilities in the context of an ongoing HRH crisis and staff shortage, which may compromise the provision of high-quality ART services. Task shifting interventions need adequate resources, relevant training opportunities, and innovative strategies to optimize the operationalization of new WHO treatment guidelines which continue to expand the number of people eligible for ART.

## Background

The rapid scale up of antiretroviral therapy (ART) for HIV and AIDS in Africa has been driven by a public health approach with emphasis on principles of decentralization, equity, patient and community participation [1]. Decentralization of ART to lower level facilities has been done to ensure long term access for rural populations and to decongest secondary and tertiary level facilities. In this context, shortage of adequate human resources for health (HRH) remains one of the major challenges [2]. According to the World Health Organisation (WHO), physician and nurse/midwife densities per 1000 population were 0.019 and 0.343 in 2009 in Malawi, 0.117 and 1.306 in 2005 in Uganda and 0.083 and 1.335 in 2011 in Zimbabwe respectively [3]. An estimation of health workforce needs for ART provision found that treating 1000 patients on ART requires about 1–2 physicians and 2–7 nurses [4].

One of the strategies to deal with staff shortages has been the shifting of tasks to lower level health cadres [5] and expert patients [6]. In Malawi, previous studies described how interventions aimed at shifting ART initiations to non-physician clinicians almost doubled ART enrolment in Thyolo district [7]. In Uganda, task shifting has been shown to reduce costs in ART follow-up [8], as nurses assumed clinical roles and lay service providers (counsellors and treatment supporters) were increasingly involved in care and support of people who are HIV-infected and affected [9]. In a cluster randomized trial in South Africa, task shifting the provision of ART from doctors to nurses was shown to be safe and led to improved health outcomes and quality of care [10].

While task shifting has been shown to increase access to ART in some resource limited settings [4], its implementation has encountered several challenges; There are concerns that its implementation may reduce the quality of care provided [11, 12]. Additional issues related to the implementation of task shifting include institutional and professional cadre resistance and maintenance of staff motivation over time [13]. In Uganda, for example, nurses felt that expert patients were a threat to their professional status due to competing training opportunities and development of close relationships between patients and expert patients resulting from expert patients’ openness about their experiences of living with HIV [14].

The Lablite project (http://www.ctu.mrc.ac.uk/our_research/research_areas/hiv/studies/lablite/) is a multi-country implementation project in Malawi, Uganda and Zimbabwe (which together account for 11% of people living with HIV globally [15]) to evaluate whether decentralized ART care can be delivered effectively at lower level health centres with limited laboratory services and to assess its economic implications. The Lablite project began with a cross-sectional survey of representative health facilities. The aim of this baseline survey was to describe and compare national and inter-country delivery of training, clinical care and use of laboratories and monitoring in health centres in national ART roll-out, providing a baseline for the project. In this paper, we compare staffing levels, turnover and perceptions and experiences of staff involved in the delivery of decentralised ART services in the three countries. We expect this paper to contribute to the boarder literature on human resources for health by illustrating the specific challenges of rolling out ART at national levels in Malawi, Uganda and Zimbabwe, through task-shifting initiatives in the context of recurrent staff shortages and limited resources.

## Methods

### Study design, data collection and analysis

A cross sectional baseline study was conducted between October 2011 and August 2012. Data were collected from a purposeful sample of 81 health facilities (20 in Malawi in 3 districts located in the northern, central and southern regions, 39 in 22 districts in Uganda, and 22 in four districts Zimbabwe). The criteria for selection of sites was to reflect a mix of rural, semi-urban and urban sites at the primary, secondary and tertiary health facility level. Facilities also included those at different stages of ART provision (including primary care facilities with no ART provision) and areas at which the Lablite project was to be implemented. The facilities were reflective of the division of service provision in these settings between the Ministries of Health (MoHs) and private/mission facilities with services agreements with the public health system, as well as the different systems of health sector decentralization. Sites were selected with the direction of Ministries of Health at the national and provincial/district levels. Primary care facilities had very limited or no research links prior to the Lablite project [16]. A mixed methods approach was used, which included a structured questionnaire and focus group discussions in order to capture data relevant to the general facility description, HRH capacity and services provided, as well as services provided at each site (HIV testing/counselling, PMTCT, ART, and laboratory services). Focus group discussions were conducted in order to complement data from the structured questionnaire. A full description of the baseline study methods has been published elsewhere [16]. Quantitative data were collected using a structured questionnaire that was administered to the staff member in charge of the facility or their representative. Data were collected on general facility description, overview of services provided, human resources for health (HRH) capacity and health care provider training. Descriptive statistical analyses were conducted stratifying by health facility level and by provision of ART services. Data are presented as medians with interquartile ranges (IQRs), or percentages as appropriate. Comparisons of catchment populations and staffing levels between ART-providing primary care health facilities and primary care health facilities with no ART provision were made using the Wilcoxon rank sum test.

Qualitative data were subsequently collected in Malawi and Zimbabwe among health care workers who were routinely providing ART services. The purpose of collecting additional qualitative data was to contextualise quantitative findings and to explore in more depth issues relating to workload, job satisfaction, training and job support. Focus groups were conducted in each health facility in order to document shared concerned and observations in relation to ART provision and workload. Participants were asked about their perceptions of workload since they started providing ART, challenges emerging as a result of ART provision, what satisfied them about their profession and their roles in ART provision. Participant were also asked about training that they have attended, trainings they would like to attend and their perceptions of access to training. We invited all health care workers who were involved in ART provision to take part in the study. We deliberately included health care workers from different cadres including nurses, medical assistants and community health workers/health surveillance assistants. In Malawi, focus group discussions (FGDs) were conducted in the local language (Chichewa), in nine primary care facilities (five providing ART, four not), three secondary care facilities and one tertiary care facility (all four providing ART). They were recorded using digital recorders, transcribed verbatim and then translated to English. In Zimbabwe, FGDs were conducted in the local language (Shona), in 19 of the 22 health facilities (14 out of 16 primary care and in five of six secondary care facilities). All the facilities were providing ART, with the majority of the primary care facilities (13/14) as outreach facilities. FGDs in Zimbabwe were not audio recorded. Instead, facilitators took detailed notes of what was discussed during the FGDs. These detailed notes were used in data analysis. As such, there were no direct quotations from the FGDs from Zimbabwe to be included in this manuscript. Data from the FGDs were analysed using content analysis [17]. Transcripts from Malawi and detailed notes from Zimbabwe were uploaded in Nvivo 8. Recurrent themes and focus group discussion questions were used to create a coding framework, on the basis of which data were fully coded.

### Ethical considerations

The study was approved by the National Health Science Research Committee in Malawi (Protocol number 889), the Joint Clinical Research Centre Institutional Review Board, the National Council for Science and Technology in Uganda (Protocol number HS 1039) and the Medical Research Council in Zimbabwe (Protocol number MRCZ/A/1630). Informed written consent was obtained from the health care workers responding to our questionnaire and those participating in focus group discussions prior to taking part in the survey. Each respondent who responded to the questionnaires or participated in focus group discussions signed two consent forms: one was given to the respondent/participant while the other was retained and kept by the research team, who safely stored signed consent forms in a locked cabinet in accordance with ethical guidelines. The consent procedure was approved by the reviewing ethics committees/research boards and forms were translated into the local languages.

## Results

Characteristics and staffing levels of all health care facilities included in the survey have been presented elsewhere [16]. Here we describe the characteristics and staffing for primary care health facilities stratifying by whether or not the facilities were providing ART services.

### Quantitative findings

#### Characteristics of primary health care facilities by ART provision

In total 53 primary care facilities were included in the survey. The majority of these facilities were located in rural areas (69% in Malawi, 71% in Uganda and 81% in Zimbabwe) (Table 1). Primary care facilities in Malawi served on average larger catchment populations than those in Uganda and Zimbabwe. At the time of the survey 30/53 facilities were providing ART (9 in Malawi, 6 in Uganda and 15 in Zimbabwe). In Malawi and Uganda, facilities that provided ART had a larger median catchment population than those who did not (Wilcoxon rank sum test for difference: *p* = .16 (Malawi); *p* = .12 (Uganda)), likely reflecting earlier ART roll-out to the larger facilities. The only site that did not provide ART in Zimbabwe was a rural health centre serving a small catchment population. Facilities in Malawi had a higher median number of patients on ART (516; IQR 241–886) compared with Uganda (206; IQR 59–660) and Zimbabwe (234; IQR 109–415). Given these findings, comparisons between countries need to consider the higher catchment populations and larger numbers of ART patients at facilities in Malawi compared with Uganda and Zimbabwe; comparisons within country need to take account of the variable catchment populations which are associated with ART provision.

**Table 1:** Facility characteristics and staffing in primary care facilities included in the baseline survey

	Malawi			Uganda			Zimbabwe		
	No ART provision	ART provision	All facilities	No ART provision	ART provision	All facilities	No ART provision	ART provision	All facilities
	*N* = 7	*N* = 9	*N* = 16	*N* = 15	*N* = 6	*N* = 21	*N* = 1	*N* = 15	*N* = 16
Location									
Urban	0 (0%)	2 (22%)	2 (13%)	1 (7%)	2 (33%)	3 (14%)	0 (0%)	2 (13%)	2 (13%)
Peri-urban	1 (14%)	2 (22%)	3 (19%)	2 (13%)	1 (17%)	3 (14%)	0 (0%)	1 (7%)	1 (6%)
Rural	6 (86%)	5 (56%)	11 (69%)	12 (80%)	3 (50%)	15 (71%)	1 (100%)	12 (80%)	13 (81%)
Catchment population^a^	29,275 (16,986–36,640)	38,286 (21,504–42,716)	29,522 (17,996–39,242)	8,600 (5,600–11,000)	39,650 (6,700–105,610)	9,000 (5,900–27,300)	4,472	9,032 (5,256–14,477)	8,616 (4,967–14,114)
Adults and children on ART		516 (241–886)			206 (59–660)			234 (109–415)	
Clinical Officers/Medical assistants									
No. staff per facility	1 (1–1)	2 (1–2)	1 (1–2)	1 (1–2)	2 (1–2)	2 (1–2)	0	0 (0–0)	0 (0–0)
No. staff per 10000 Catchment	.34 (.25–.59)	.46 (.26–1.0)	.38 (.26–.92)	1.20 (.91–2.45)	.56 (.19–1.49)	1.16 (.72–2.22)	0	0 (0–0)	0 (0–0)
Midwives/nurses									
No. staff per facility	2 (1–3)	3 (2–13)	2.5 (1–4.5)	3 (2–6)	7 (4–12)	4 (3–6)	3	6 (2–15)	6 (2.5–14)
No. per 10000 Catchment	.76 (.27–1.81)	.70 (.52–1.73)	.73 (.40–1.77)	3.67 (1.06–8.70)	1.89 (.71–11.94)	3.64 (1.06–8.70)	6.7	4.5 (2.8–8.7)	4.9 (2.8–8.2)
Counsellors									
No. staff per facility	0 (0–0)	0 (0–0)	0 (0–0)	0 (0–0)	0 (0–0)	0 (0–0)	0	1 (1–1)	1 (1–1)
Auxiliary staff									
No. staff per facility	5 (4–9)	5 (3–6)	5 (3.5–7.5)	2 (0–3)	2.5 (0–3)	2 (0–3)	5	3 (3–9)	3 (3–9)
Community health workers									
No. staff per facility	11 (8–20)	19 (17–26)	18 (11–22)	0 (0–0)	0 (0–1)	0 (0–0)	4	0 (0–0)	0 (0–0)
Administrative staff									
No. staff per facility	0 (0–3)	0 (0–1)	0 (0–2)	0 (0–0)	0.5 (0–1)	0 (0–1)	0	0 (0–0)	0 (0–0)
No. facilities with ≥1 admin staff	3 (43%)	4 (44%)	7 (44%)	3 (20%)	3 (50%)	6 (29%)	0 (0%)	3 (20%)	3 (19%)
Laboratory staff									
No. staff per facility	0 (0–1)	0 (0–1)	0 (0–1)	1 (0–1)	0.5 (0–1)	1 (0–1)	0	0 (0–0)	0 (0–0)
No. facilities with ≥1 lab staff	2 (29%)	4 (44%)	6 (38%)	11 (73%)	3 (50%)	14 (67%)	0 (0%)	1 (7%)	1 (6%)

Values are n (col %) or median (IQR)

^a^Size of the population served by the healthcare facility

#### Staffing in primary health care facilities by ART provision

All but one primary care facility in Malawi and one in Uganda were led by a non-physician clinical officer/medical assistant; primary care facilities in Zimbabwe were led entirely by nurses/midwives (Table 1). In Malawi, numbers of clinical officers/medical assistants were higher in the facilities with ART provision; 6/9 facilities with ART provision had 2 or more staff of this cadre whereas 6/7 facilities with no ART provision had 1 clinical officer/medical assistant and 1/7 had 0 (test for difference, *p* = 0.01). However, per 10,000 catchment population staffing was similar (median (IQR) clinical officers/medical assistants 0.46 (0.26 - 1.0) in facilities with ART provision compared with 0.34 (0.25–0.59) in facilities with no ART provision (*p* = 0.22). In Uganda 4/6 facilities with ART provision had 2 clinical officers/medical assistants compared with 7/15 facilities with no ART provision (the remainder had 1, test for difference *p* = 0.4). Staffing was also similar in Ugandan facilities with and without ART provision after adjusting for catchment population (*p* = 0.1).

Nurse/midwife densities per 10,000 catchment population were higher in Uganda and Zimbabwe (median 3.6 and 4.9 respectively) than in Malawi (0.7). In Malawi and Uganda nurse/midwife densities were similar in facilities with and without ART provision (*p* = 0.1; *p* = 0.6 respectively). In Malawi, facilities with and without ART provision had community health workers. These were not available in Uganda. There were four (4) community health workers in the primary care facility that was not providing ART in Zimbabwe.

In Malawi and Uganda, about half of the facilities that were providing ART had at least one laboratory technician/assistant. In Zimbabwe where ART provision was predominantly by a hospital outreach team, only one facility among the 15 included in the survey that were providing ART, had a laboratory technician/assistant. Few facilities had any administrative staff; among facilities providing ART only 44%, 50% and 20% of facilities in Malawi, Uganda and Zimbabwe had an administrative staff member.

In summary, levels of staffing (particularly by cadre) differed greatly between countries but were low in all three settings; facilities with ART provision tended to be the larger healthcare facilities and once this was allowed for there was little suggestion of increased staffing levels to provide ART services.

#### Staff turnover in all health care facilities

Table 2 describes staff turnover by cadre in the 3 months prior to survey and estimated annual turnover (assuming that the 3 months turnover is maintained over the year). With the qualitative data presented below, these data are an indication of staff morale in the facilities. In each of the three countries, between 10 to 20% of all health care facilities saw at least one clinical officer/medical assistant leave in the 3 months prior to the survey (Table 2). In primary care facilities, projected annual turnover of clinical officers/medical assistants was 21%. In secondary care facilities, the projected annual turnover was 13% in Malawi and Uganda, and 67% in Zimbabwe. By facility level and country, the projected turnover of nurses/midwives was higher than turnover in clinical officers/medical assistants. In primary care facilities, projected annual turnover of nurses/midwives were 32%, 19% and 5% in Malawi, Uganda and Zimbabwe respectively. Projected turnover, albeit estimated from a snapshot of data over 3 months, was high in general, suggesting likely staff shortages, low staff morale and costs to the health care systems.

**Table 2:** Staff turnover in all health facilities included in the baseline survey

	Malawi			Uganda			Zimbabwe	
	Primary care	Secondary care	Tertiary care	Primary care	Secondary care	Tertiary care	Primary care	Secondary care
	*N* = 16	*N* = 2^a^	*N* = 1^b^	*N* = 21	*N* = 16	*N* = 2	*N* = 16	*N* = 6
Physicians								
Facilities where ≥ 1 staff left in 3 months	NA	0/1^c^		NA	1/3	½	NA	0/5
3 month turnover over all facilities	NA	0/2^c^		NA	1/45	9/20	NA	0/5
Estimated annual turnover over all facilities	NA	0%		NA	8.9%	180%	NA	0%
Clinical Officers/Medical assistants								
Facilities where ≥ 1 staff left in 3 months	2/15	1/2		0/21	3/16	½	NA	1/5
3 month turnover over all facilities	2/38	2/60		0/32	3/93	1/37	NA	1/6
Estimated annual turnover over all facilities	21%	13%		0%	13%	11%	NA	67%
Midwives/nurses								
Facilities where ≥ 1 staff left in 3 months	5/14	2/2	1/1	5/21	6/16	2/2	1/16	3/6
3 month turnover over all facilities	6/74	3/90	3/317	5/107	23/596	14/197	2/156	4/114
Estimated annual turnover over all facilities	32%	13%	4%	19%	15%	28%	5%	14%
Auxiliary staff								
Facilities where ≥ 1 staff left in 3 months	2/15	0/2		5/14	1/11	0/2	0/16	2/6
3 month turnover over all facilities	2/97	0/137		6/42	1/161	0/113	0/98	3/391
Estimated annual turnover over all facilities	8%	0%		57%	2.5%	0%	0%	3%
Administrative staff								
Facilities where ≥ 1 staff left in 3 months	1/7	0/2		0/6	1/12	2/2	0/3	0/6
3 month turnover over all facilities	1/17	0/14		0/6	1/91	2/22	0/8	0/73
Estimated annual turnover over all facilities	24%	0%		0%	4.4%	36%	0%	0%
Laboratory staff								
Facilities where ≥ 1 staff left in 3 months	0/6	1/2		0/14	0/15	0/2	0/1	0/6
3 month turnover over all facilities	0/8	1/9		0/17	0/44	0/17	0/1	0/18
Estimated annual turnover over all facilities	0%	44%		0%	0%	0%	0%	0%

Facilities where ≥ 1 staff left in 3 months provides number of facilities with ≥ 1 member of staff of respective cadre leaving in 3 month period before the survey; the denominator is the number of facilities with any staff over the 3 month period of that cadre (so is less than n if one or more facilities had no staff at that level)

Three month turnover is the total number of staff members of a respective cadre leaving in the 3 month period across all facilities with the denominator equal to the number of staff employed at that level (including those who left but not replaced)

The annual estimated turnover assumes that the 3 month turnover is maintained for a year

^a^No data were available for one secondary care facility in Malawi except for physicians (4 physicians were in post and none had left); this facility is excluded throughout for consistency

^b^Data were available on nurses/midwives only for the tertiary care facility in Malawi

^c^Data missing on physicians for second facility

*NA* not applicable because no staff at this level

### Qualitative findings

#### Perceptions of staff shortages

Data collected during FGDs with HCWs show similarities between HCWs’ perceptions around staff shortage in Malawi and Zimbabwe. HCWs in both ART facilities and non-ART facilities said there was shortage of staff for all services within their respective health facilities, even before ART services were introduced.

“*It is not that the shortage has come as a result of the introduction of these services but the staff shortage has existed all along*”. (FGD, Primary care non-ART site, Malawi).

In some cases, HCWs perceived the shortage in terms of staff that were trained to provide a particular service.

“*According to my opinion the biggest challenge is the shortage of staff*… *If there was an opportunity it would have been better if more staff were trained in HIV testing and counselling*, *it could have been better in alleviating the problem of shortage of staff*”. (FGD, Primary care ART site, Malawi).

“*Moreover*, *when it comes to nurses*, *I am the only nurse here who was trained in HTC but in total*, *there are four nurses at this health facility. When the counsellors and I are not available*, *the other nurses cannot offer the HTC services and they wait for the counsellors and if they will not come*, *it means on that day the people will not be tested*”. (FGD, Primary care non-ART site, Malawi).

For some HCWs in Malawi and Zimbabwe, shortage of staff was linked to perceived high staff turnover rates as a result of poor working conditions, including low pay and lack of training opportunities. This involved staff leaving rural health facilities to move to urban facilities and/or staff leaving government health facilities to join the private health facilities or non-governmental organizations, both of which are perceived to be financially more beneficial than government-owned health facilities.

#### Increased workload in the context of shortage of staff

The introduction of ART was perceived by HCWs to have increased workload. For example the required administrative burden that came with ART provision was one of the areas HCWs perceived as an increase in workload. In non-ART sites that were providing PMTCT, HCWs also said their workload had increased. They perceived these as new services which are supposed to be provided on top of their ‘normal’ day to day duties.

In Malawi, the implementation of Option B+ from July 2011 meant that all pregnant women and lactating mothers who test positive are eligible for lifelong ART. This has increased the number of people who are on ART. Some HCWs said this had significantly increased their workload.

“*The introduction of new regimen has made more people to access ART* […] *and having a lot of people also means a lot of work for the providers*…”. (FGD, Tertiary Care ART site, Malawi).

HCWs perceived that the increase in workload has had several consequences. Some felt that the increase in workload has resulted in extending their working hours. One health care worker said:

“*We extend our working hours beyond the contractually agree time for knocking off*, *the amount of overtime hours per day has been increasing because for us to manage these people properly we need more time to attend to them in our respective consultation rooms*”. (FGD, Secondary Care ART site, Malawi).

Other HCWs felt that the increase in number of patients is leading to overcrowding in health facilities and hence hindering the provision of the *quality* of service. For others, quality of service provision was also affected by lack of equipment and drug stock-outs.

#### “The job is like a calling from God”: perceptions of low job satisfaction

Most of the participants in the FGDs highlighted that they were not satisfied with their work. There were several reasons that were mentioned as contributing to these feelings of dissatisfaction.

Most HCWs felt that their salaries were low in relation to their workload. They perceived that their workload had substantially increased, resulting in working longer hours while their monthly income had not.

“*What I can say is that due to an increase in workload on the already limited staff*, *we indeed work over time and there is no time to rest. Further to this*, *after working for long periods of time*, *we do not get any allowance as compensation for that*”. (FGD, Primary care non-ART site, Malawi).

Those who took on more technical tasks as part of task shifting, for example Health Surveillance Assistants (HSAs) in Malawi, said that they should have had a salary increment as a result of assuming these new roles. With the introduction of ART more specifically, HCWs felt that their salaries should have increased as their workload is considerably greater (ART is perceived as an additional responsibility to the role of community health workers, clinicians, nurses and medical assistants).

“[…] *I am an HSA by profession*, *my job as HTC counsellor is just supplementary but we are not given top up incentives to feel motivated when working*” (FGD, Primary Care ART Site, Malawi).

A pervasive perception of unfair and inequitable access to training opportunities were also mentioned as a source of low job satisfaction. HCWs, especially those in rural facilities and in lower level positions, felt that opportunities for training were exclusively given to higher cadres of HCWs, or only available for urban and district staff rather than for those in rural areas.

Some HCWs felt that they have not undergone enough relevant training in order to care for ART patients. Increasingly, they are dealing with clinical tasks for which they feel unequipped.

“*Sometimes we are asked to test a mentally sick person and yet we have never learned on how to handle psychiatric patients*-*we are forced to do a quick job and give results. So we are denied chances* [*to take part*] *in most of the trainings and yet we meet different issues which need trained personnel to handle*”. (FGD, Secondary Care ART site, Malawi).

Some HCWs also stressed that they needed refresher training as “things change”. Lack of training opportunities resulted in reduced confidence in HCWs conducting their duties:

“*I don*’*t get satisfied with the job that I do. There are two reasons*; *one is the issue of low salary and secondly I feel undermined because of the fact that I don*’*t know how to give all HIV*-*related services while the other people doing the same work are professionals in the field. It becomes difficult for me to refer patients to other people who are working in the same department as me*”. (FGD, Primary Care non-ART site, Malawi).

Other issues that contributed to perceptions of low job satisfaction for HCWs included a lack of clear career advancement opportunities and inadequate resources and infrastructure for quality service provision.

## Discussion

This cross sectional study has highlighted some of the HRH challenges in the provision of ART in Malawi, Zimbabwe and Uganda. Despite the introduction of ART and its rapid scale-up in each country, staffing levels were not increased to cater for these new and specialized services. Our quantitative and qualitative data reveal a serious shortage of staff. In the context of inadequate equipment and infrastructure, this contributed to low job satisfaction and motivation. Across the three countries, staff turnover was high, especially among nurses and clinical officers. High staff turnover, inadequate equipment and low job satisfaction have significant implications in the ART service provision across the three countries. Our qualitative data show that HCWs perceived an increase in workload with the introduction of ART provision, and felt that staffing levels should have been increased given the additional work burden. Apart from recruitment of new staff, participants also felt the need to train staff who were already at the facilities as few of them had undergone ART related trainings. Data also show that some HCWs mentioned having low job satisfaction due to a perception of an inadequate salary in relation to their actual workload and due to the extended working hours in the context of ART delivery.

The implementation of task shifting as a strategy to deal with shortage of staff is based on the principle of delegation “of medical and health service duties from higher to lower cadres or new cadres” [13]. In Malawi, health surveillance assistants (HSAs or community health workers) and expert patients have taken on some of the tasks previously performed by nurses (including distribution of drugs, HIV testing and counselling). However, lower level cadres of HCWs, who are now assuming higher cadre duties, feel inadequately remunerated in relation to the more specialized (and additional) tasks which they are required to perform. Our study showed that in many instances, task shifting has occurred in the context of limited additional resources, existing shortage of staff, high levels of staff turnover and lack of adequate equipment. As such, some tasks were shifted to lower level cadres prior to having undergone requisite training. Inequitable access to training has been noted elsewhere as a source of demotivation among health care workers [18]. Our data indicate that this was linked to availability of financial incentives during training. All these have raised concerns that task shifting may reduce that quality of ART services provided [12].

A shortage of staff has resulted in some services not being implemented at lower level health facilities. In most primary care facilities, laboratory monitoring of ART patients does not usually take place [16]. This is partly due to lack of trained laboratory technicians and/or lack of reagents.

Since the baseline study was conducted, there have been changes in policy across the three countries. In 2014, the Malawi ART guidelines were revised to include routine viral load monitoring and raising of the CD4 threshold for initiation on ART [19]. Uganda and Zimbabwe have since also adopted Option B+ as a strategy for provision of ART. A review of Option B+ implementation in 11 countries has shown challenges with regard to staffing, with introduction of new cadres in some countries [20]. As HIV treatment guidelines change to provide for access to ART for people living with HIV with lower CD4 cell counts and Option B+ is rolled out in all three countries, the potential impact of task shifting on provision of quality services is even greater. While the 2013 World Health Organization guidelines on antiretroviral drug use for treating and preventing HIV [21] recommend regular training, mentoring and supervision of HCWs to ensure high quality care and implementation of updated national recommendations, this may not be enough in some contexts. As countries are moving towards universal treatment for HIV, such problems will be further exacerbated as more people are initiated on ART. The findings from this study highlight the need for more resources to be directed towards improving health care worker morale, job satisfaction and ensuring equitable access to training opportunities to reduce staff turnover and ensure provision of optimal ART services.

### Limitations of the study

This was a cross sectional study on a purposive sample of 81 facilities in the three countries. As such, the findings cannot be generalised to the rest of the facilities in the three countries. The cross sectional design means that changes (particularly in response to the introduction of ART services) were not captured at a facility level. The small numbers of facilities means there is low power to formally compare facilities within countries. Qualitative data was not collected in Uganda due to logistical limitations. As such, there are no comparisons across the three countries based on qualitative data. Direct quotations from Zimbabwe are not included in this paper because focus group discussions were not recorded verbatim; instead, facilitators of focus group discussions systematically summarised notes of the discussions in Zimbabwe. The data from Zimbabwe were included in the analysis however we recognise that the conclusions derived from the qualitative data requires further investigation. In this study, we collected facility level data on staffing. We did not collect individual level data number of working hours, overtime and workload. Further to this, there have been changes in ART policy guidelines in the three countries since the baseline study was done. With such changes, some of the issues raised in this paper may have changed as well.

## Conclusions

Challenges of staffing, inadequate training and low salaries have already been noted in other countries in Sub-Saharan Africa. In rural primary care facilities, decentralization of ART has exacerbated these problems and hence compromised the provision of some ART services. While task shifting has been implemented to deal with staff shortages, it is not enough. In addition to HRH planning and forecasting, optimization of existing frontline HCWs is critical for supporting the increasing numbers of ART eligible patients with operationalization of the WHO 2013 HIV Treatment guidelines which will continue to expand the number of people eligible for HIV treatment.

## Abbreviations

ART: Antiretroviral therapy
FGD: Focus group discussion
HCW: Health care worker
HRH: Human resources for health
MoH: Ministry of health
PMTCT: Prevention of mother to child transmission
WHO: World health organisation
